# Supplementary material for: Spontaneous redox continuum reveals sequestered technetium clusters and retarded mineral transformation of iron
Source: Commun Chem. 2020 Jul 10;3:87. doi: 10.1038/s42004-020-0334-x (PMC9814752; doi:10.1038/s42004-020-0334-x)
Supplement: Supplementary file 1 — Supplementary Information [file 42004_2020_334_MOESM1_ESM.pdf]

Supplementary Information

Spontaneous Redox Continuum Reveals Sequestered Technetium Clusters and Retarded Mineral Transformation of Iron

Daria Boglaienko[†], Jennifer A. Soltis[†], Ravi K. Kukkadapu[†], Yingge Du[†], Lucas E. Sweet[†], Vanessa E. Holfeltz[†], Gabriel B. Hall[†], Edgar C. Buck[†], Carlo U. Segre[‡], Hilary P. Emerson[†], Yelena Katsenovich[§], Tatiana G. Levitskaia[†*]

[†]Pacific Northwest National Laboratory

[‡]Illinois Institute of Technology

[§]Florida International University

[*] Corresponding author: Tatiana.Levitskaia@pnnl.gov

Supplementary figures

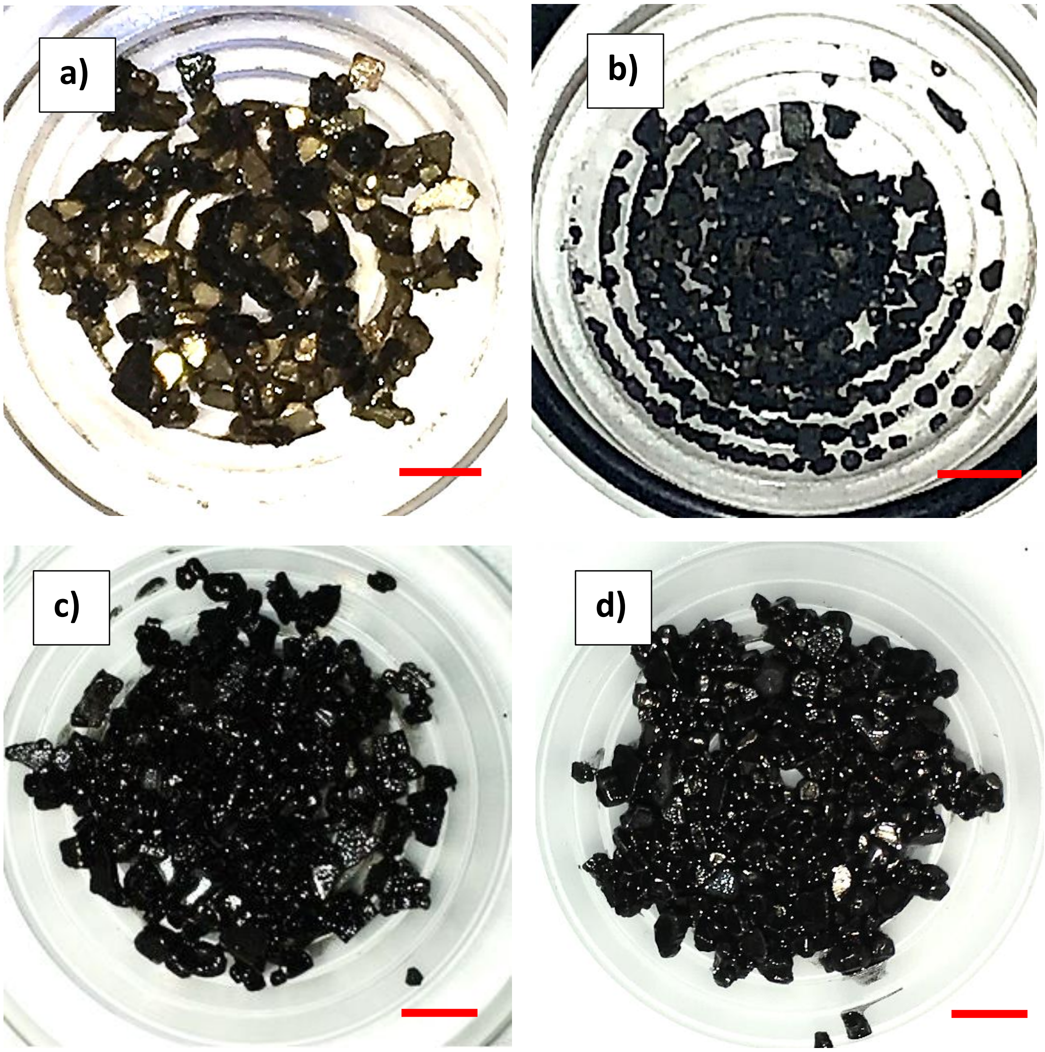

Supplementary Figure 1. Photographs of granular ZVI contacted with and without 17 mM of TcO_4^- in 80 mM NaCl: **a)** ZVI reacted with Tc for one week; **b)** ZVI reacted with Tc for one month; **c)** ZVI reacted without Tc for one week; **d)** ZVI reacted without Tc for one month. Scale bar: 4 mm.

a)

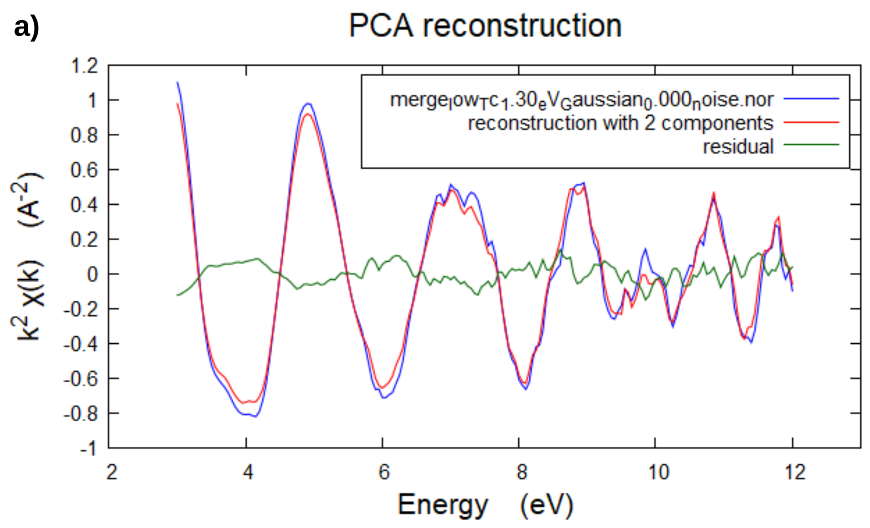

b)

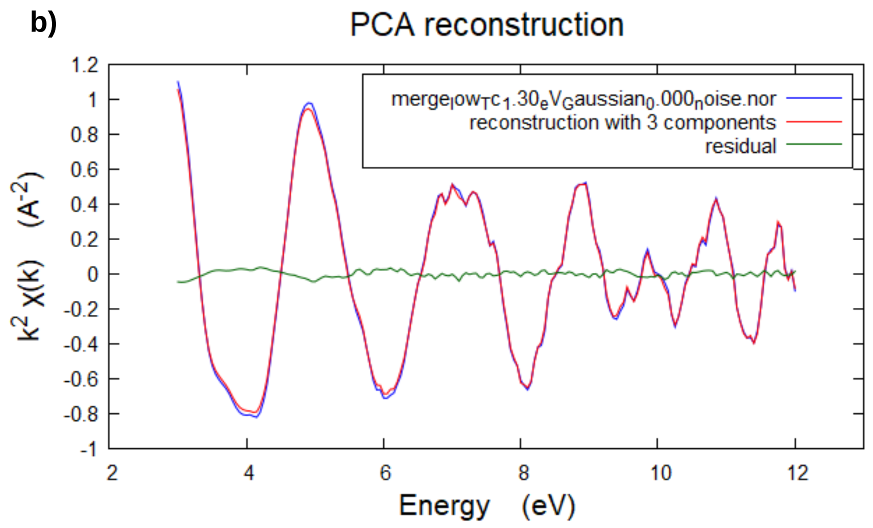

Supplementary Figure 2. Principal component analysis (PCA) for the EXAFS region of the spectra ($3 < k < 12$) and reconstruction with two components (**a**) and three components (**b**).

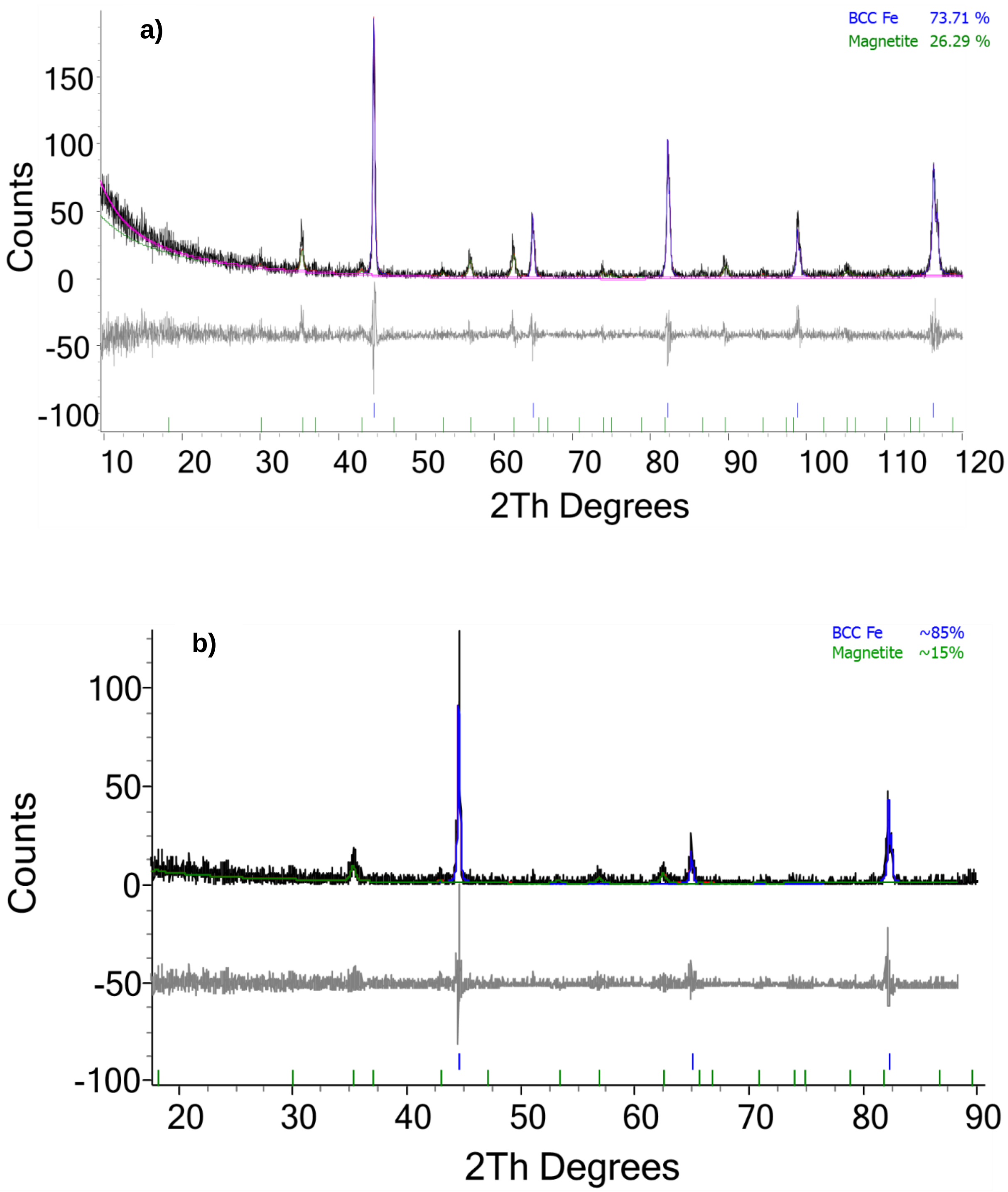
a)
BCC Fe 73.71 %
Magnetite 26.29 %
Counts
2Th Degrees
b)
BCC Fe ~85%
Magnetite ~15%
Counts
2Th Degrees

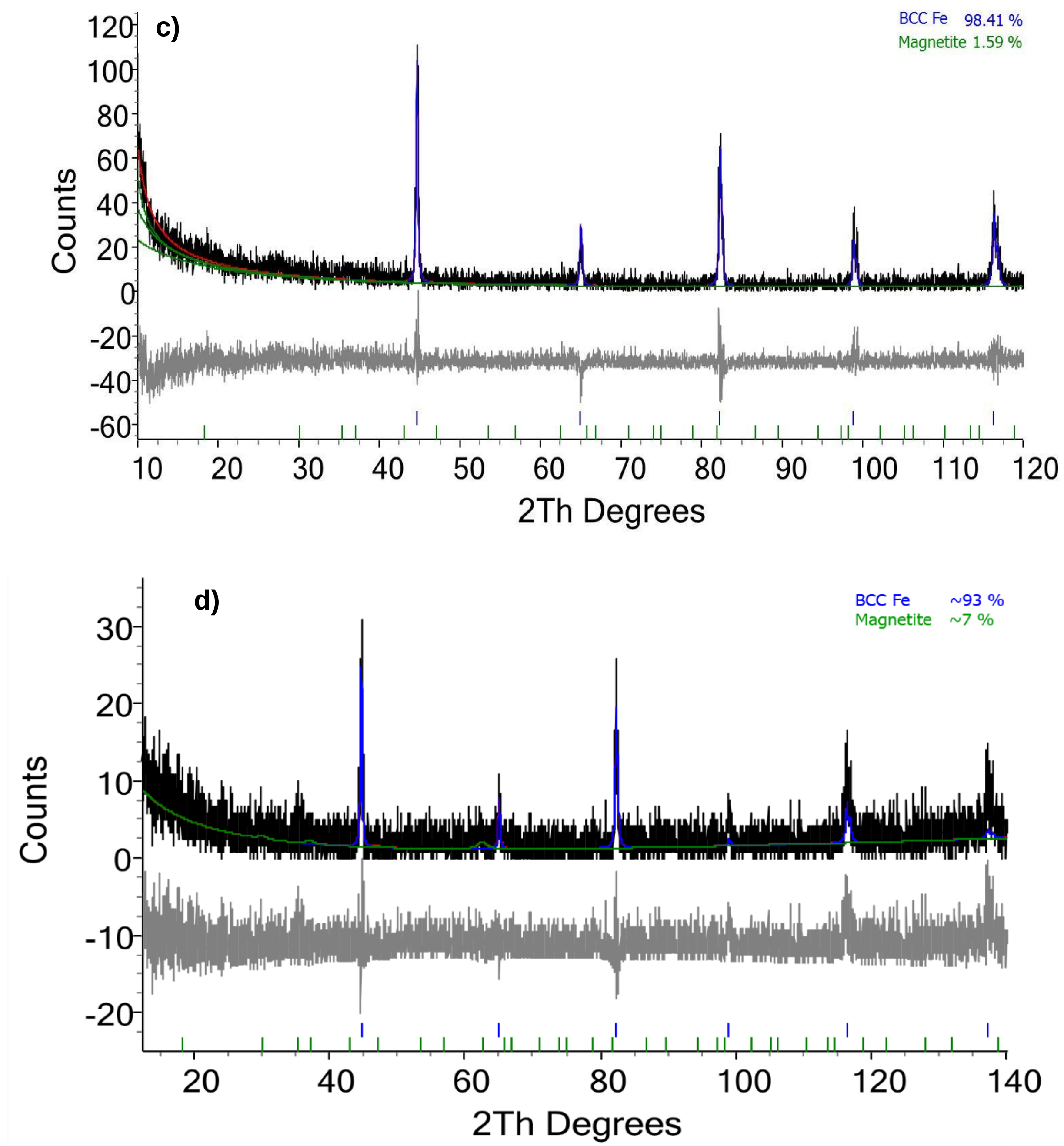

Supplementary Figure 3. PXRD patterns for granular ZVI reacted with and without 17 mM TcO_4^- in 80 mM NaCl: **a)** ZVI reacted without Tc for one week; **b)** ZVI reacted without Tc for one month; **c)** ZVI reacted with Tc for one week; **d)** ZVI reacted with Tc for one month.

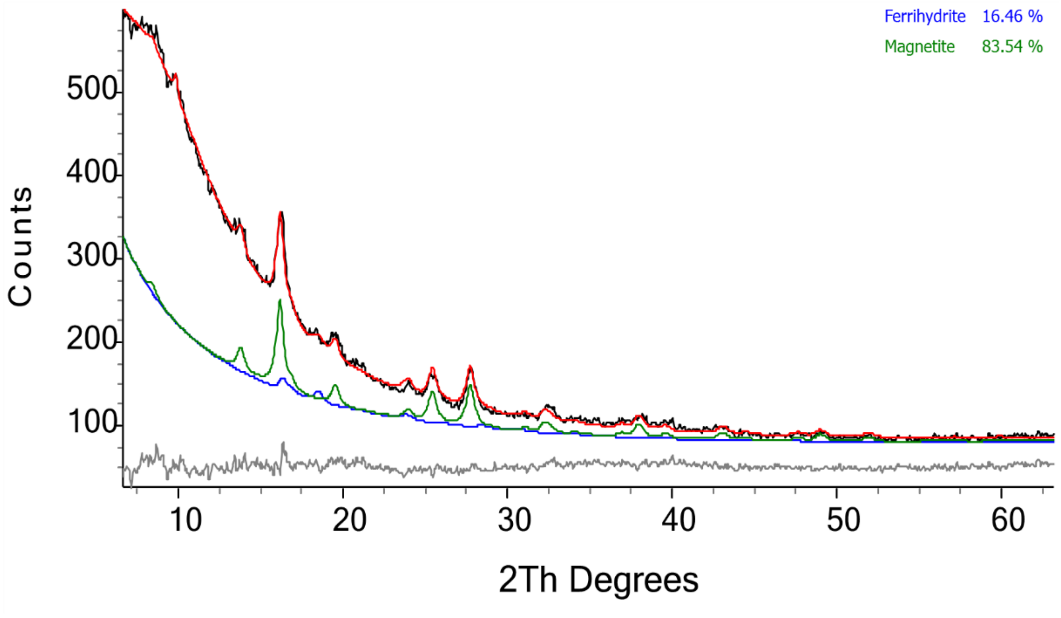

Supplementary Figure 4. Diffractogram of granular ZVI reacted in 80 mM NaCl for one month using a Bruker D8 Venture single crystal diffractometer with Mo k_α radiation.

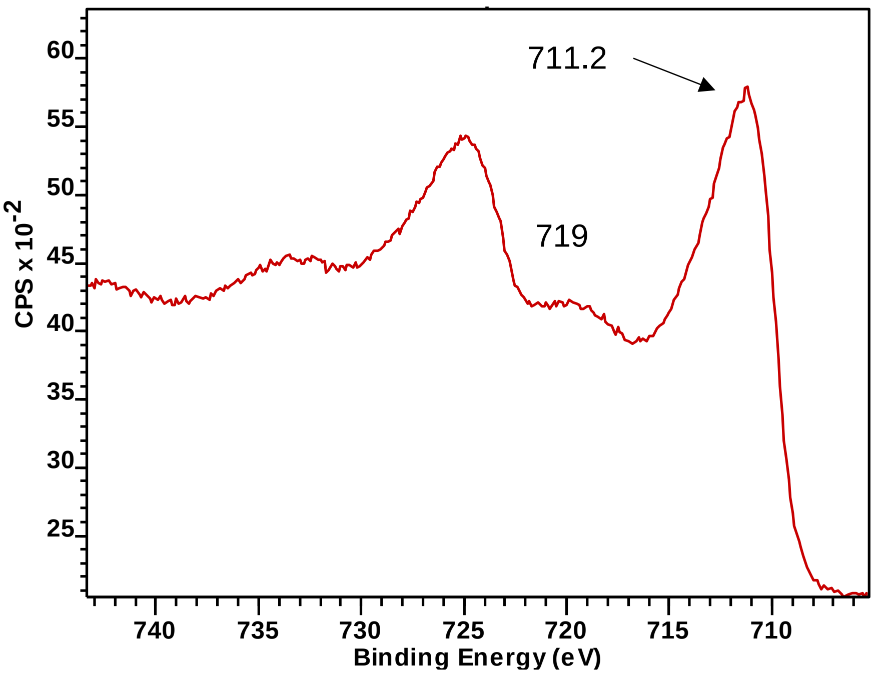

Supplementary Figure 5. XPS Fe 2p spectra for granular ZVI contacted with 17 mM TcO_4^- in 80 mM NaCl for one month.

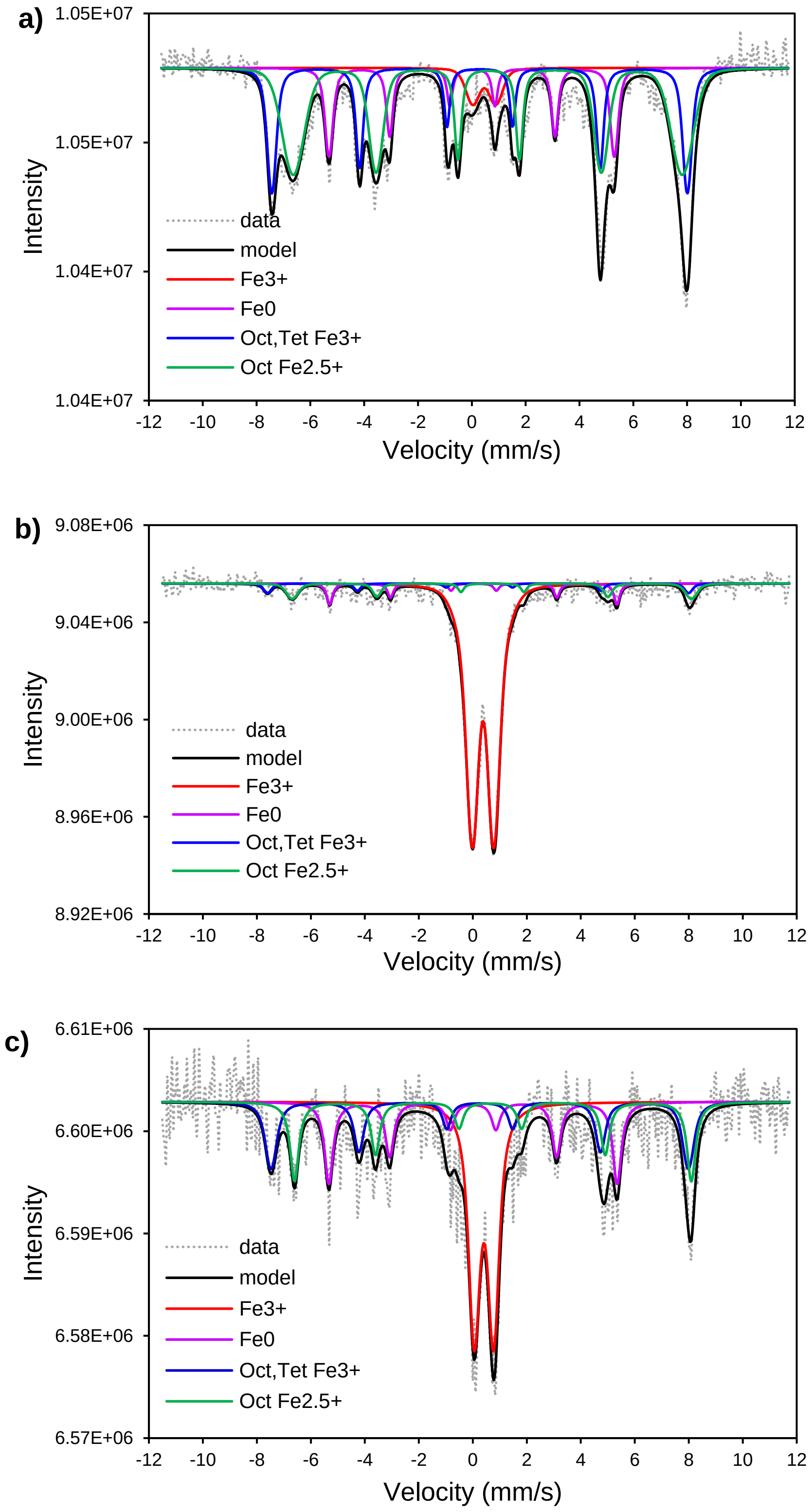

Supplementary Figure 6. 298 K Mössbauer spectra for granular ZVI reacted with and without 17 mM TcO_4^- in 80 mM NaCl: **a)** ZVI reacted without Tc for one week; **b)** ZVI reacted with Tc for one week; **c)** ZVI reacted with Tc for one month.

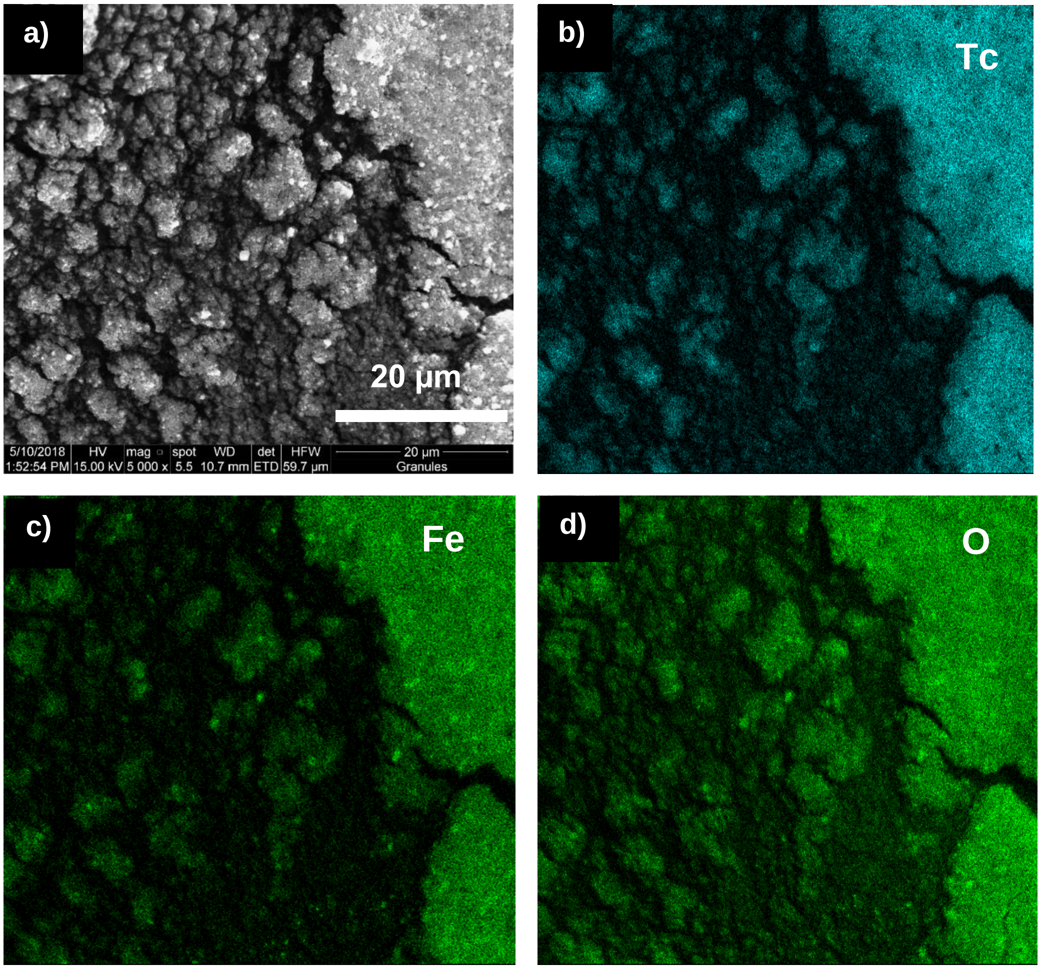

Supplementary Figure 7. SEM and EDS images of the granular ZVI reacted with 17 mM TcO_4^- in 80 mM NaCl for one week: **a)** SEM micrograph of the surface of oxidized granules; **b)-d)** EDS maps of Tc (b), Fe (c), and O (d).

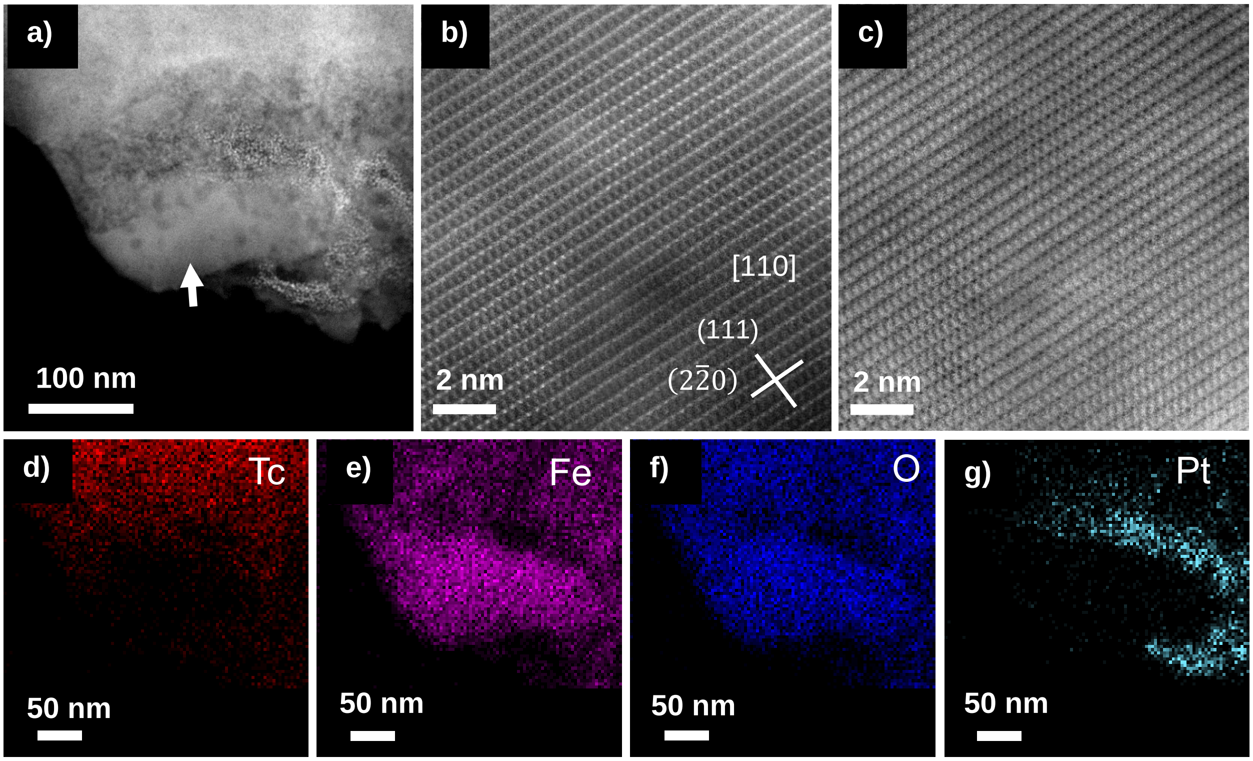

Supplementary Figure 8. STEM and EDS nano scale images of the granular ZVI reacted with 17 mM TcO_4^- in 80 mM NaCl for one month with the region of absence of Tc. **a)** HAADF STEM image of ZVI reacted with Tc; **b)** and **c)** atomic resolution images of the area indicated with a white arrow in (a) show magnetite or maghemite structure imaged along the [110] zone axis. These images were collected concurrently with (b) HAADF and (c) bright field detectors. **d)-g)** EDS maps of (a) showing Tc (d), Fe (e), O (f), and Pt (g) overlaid onto the electron image. These images indicate the presence of an iron oxide phase (magnetite or maghemite) that does not have significant Tc content. The small bright spots in (a) are Pt. Arrow indicates the area imaged at higher resolution in (b) and (c).

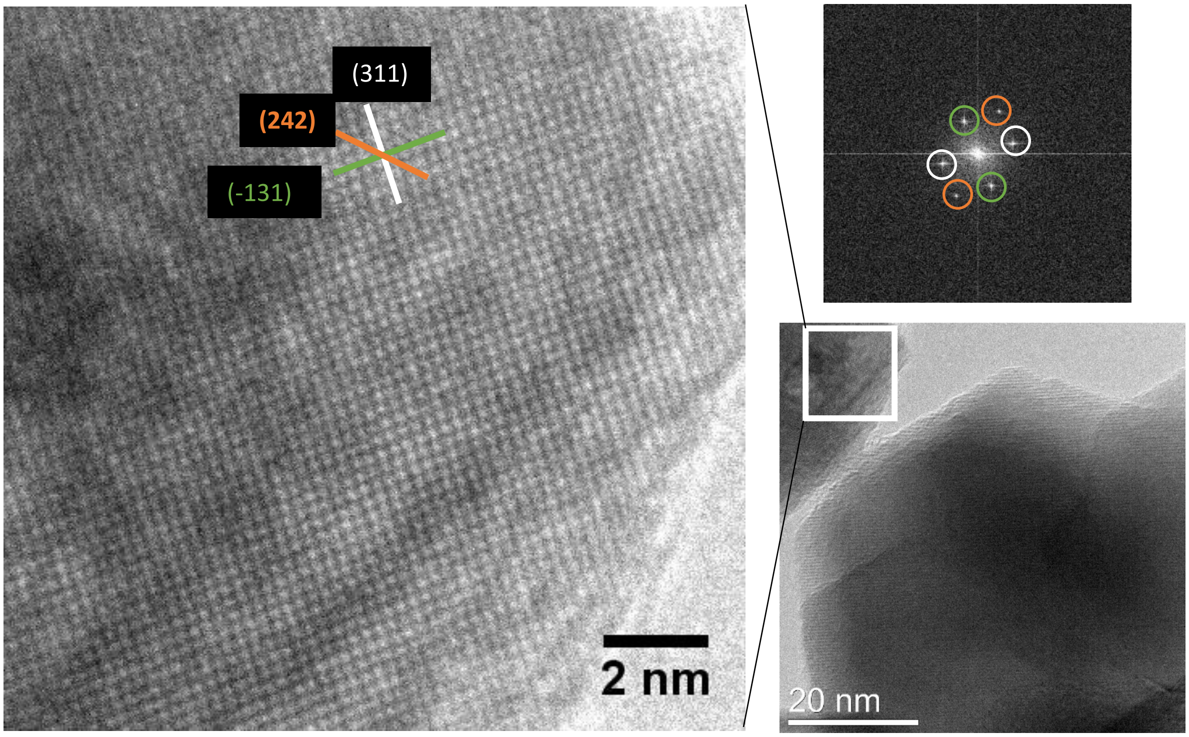

Supplementary Figure 9. STEM nano scale images of granular ZVI reacted in 80 mM NaCl for one month (without Tc).

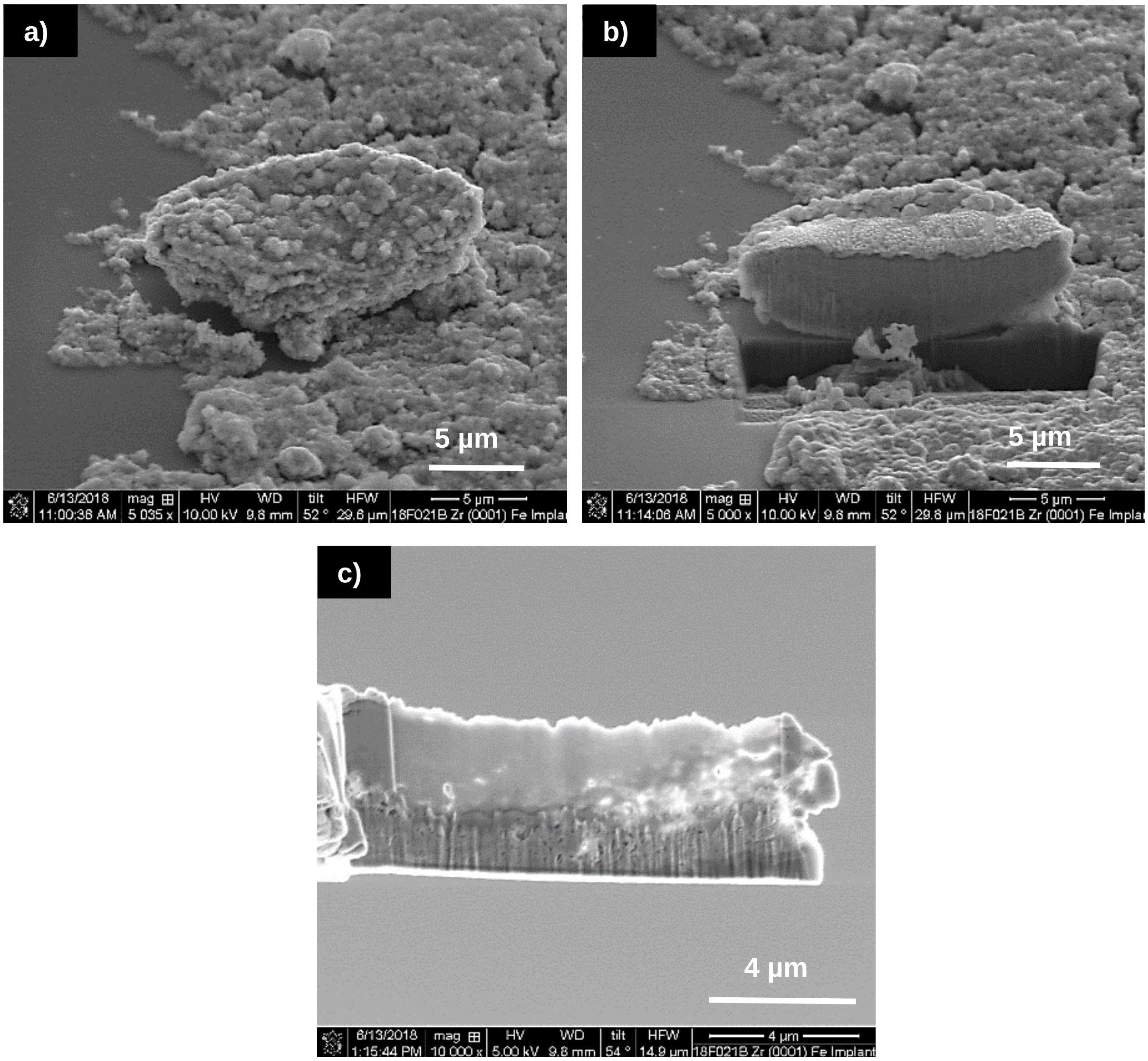

Supplementary Figure 10. SEM images of granular ZVI contacted with 17 mM TcO_4^- in 80 mM NaCl for one month. **a)** The granule of ZVI chosen for FIB liftout; **b)** the liftout in progress; **c)** the thinned liftout that was imaged by STEM.

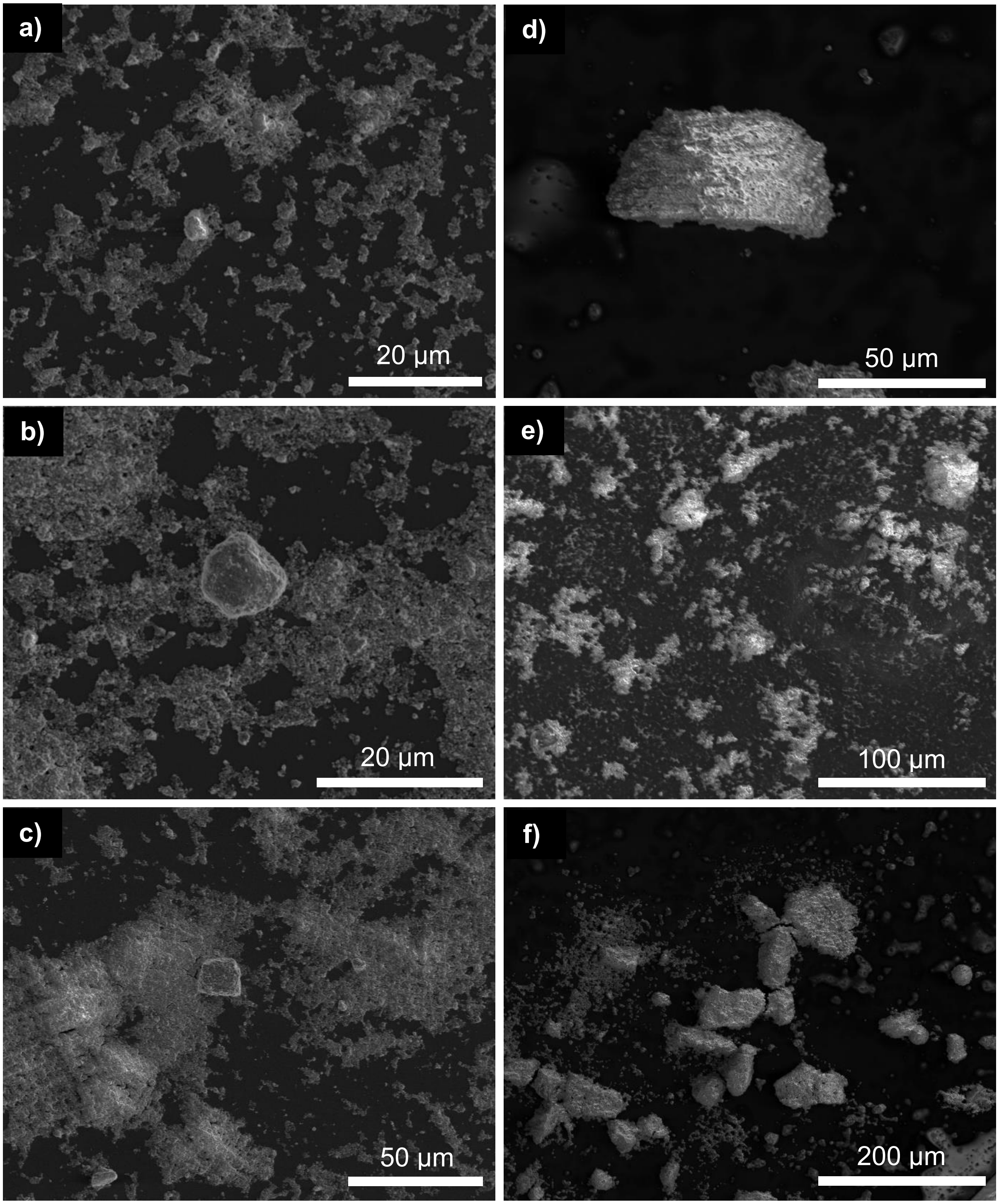

Supplementary Figure 11. Scanning electron micrographs of granular ZVI contacted with and without 17 mM TcO_4^- in 80 mM NaCl for one month that show the heterogeneity of the iron oxide granules. Left column: **a)-b)** ZVI reacted with Tc; right column: **d)-f)** ZVI without Tc.

Supplementary tables

Supplementary Table 1. Linear combination fit (LCF) combinatorics for the EXAFS region of the spectra (3 < k < 12).

Standards:<br><br>A – Tc^{4+} in Fe_3O_4<br>B – TcO_2•nH_2O<br>C – TcO_4^-	R-factor	Chi_r^2
A, B, C	0.38796	0.0472
A, B	0.63044	0.0763
B, C	0.65905	0.0798
A, C	0.81133	0.0983

Supplementary Table 2. Results of Mossbauer spectroscopy of the granular ZVI oxidized in the presence and absence of Tc for the short and long term.

	ZVI without Tc		ZVI with Tc		
Oxidation time	one week	one month	one week[a]	one month	one month[b]
Mineral phase, %	Fe^0 17.8<br>Oct $Fe^{2.5+}$ 48.9<br>Oct,Tet Fe^{3+} 27.9<br>Fe^{3+} 5.4	Fe^0 10.4<br>Oct $Fe^{2.5+}$ 46.4<br>Oct,Tet Fe^{3+} 36.5<br>Fe^{3+} 6.8	Fe^0 7.0<br>Oct $Fe^{2.5+}$ 8.8<br>Oct,Tet Fe^{3+} 4.1<br>Fe^{3+} 80.1	Fe^0 21.5<br>Oct $Fe^{2.5+}$ 20.8<br>Oct,Tet Fe^{3+} 21.3<br>Fe^{3+} 36.4	Fe^0 17.7<br>Oct Fe^{2+} 22.0<br>Oct,Tet Fe^{3+} 33.0<br>Fe^{3+} 27.3
Fe^{2+}/Fe^{3+}	0.47	0.39	0.52	0.33	0.25

[a] sample was analyzed for the finest particle fraction mainly, excluding large granules due to poor signal, which caused relatively low value for Fe^0;

[b] additional spectra taken at 77 K for the same one-month contact sample.

Supplementary Table 3. Alternative EXAFS fit to test a model with multiple scattering pathways in TcO_2.

Scan location	Path	CN	R (Å)	σ^2 (Å^2)	ΔE_0 (eV)	Chi_r^2	R-factor
**ZVI-2-B**	Tc-O	6	1.98	0.012	-3.1	2091	0.12
	Tc-Tc	1	2.59	0.004			
	Tc-O-Tc	4	3.25	-0.005			
	Tc-O-O	4	-2.28	2.638			
